# Defining early changes in Alzheimer’s disease from RNA sequencing of brain regions differentially affected by pathology

**DOI:** 10.1038/s41598-021-83872-z

**Published:** 2021-03-01

**Authors:** Boris Guennewig, Julia Lim, Lee Marshall, Andrew N. McCorkindale, Patrick J. Paasila, Ellis Patrick, Jillian J. Kril, Glenda M. Halliday, Antony A. Cooper, Greg T. Sutherland

**Affiliations:** 1 Brain and Mind Centre and School of Medical Sciences, Faculty of Medicine and Health, The University of Sydney, Camperdown, NSW 2006 Australia; 2 Discipline of Pathology and Charles Perkins Centre, School of Medical Sciences, Faculty of Medicine and Health, Rm 6211 Level 6W, The University of Sydney, Sydney, NSW 2006 Australia; 3 St. Vincent’s Clinical School and School of Biotechnology and Biomolecular Sciences, University of New South Wales, Kensington, NSW 2052 Australia; 4 Garvan Institute of Medical Research, Darlinghurst, NSW 2010 Australia; 5 School of Mathematics and Statistics, Faculty of Science, The University of Sydney, Camperdown, NSW 2006 Australia

**Keywords:** Alzheimer's disease, Alzheimer's disease

## Abstract

Tau pathology in Alzheimer’s disease (AD) spreads in a predictable pattern that corresponds with disease symptoms and severity. At post-mortem there are cortical regions that range from mildly to severely affected by tau pathology and neuronal loss. A comparison of the molecular signatures of these differentially affected areas within cases and between cases and controls may allow the temporal modelling of disease progression. Here we used RNA sequencing to explore differential gene expression in the mildly affected primary visual cortex and moderately affected precuneus of ten age-, gender- and RNA quality-matched post-mortem brains from AD patients and healthy controls. The two regions in AD cases had similar transcriptomic signatures but there were broader abnormalities in the precuneus consistent with the greater tau load. Both regions were characterised by upregulation of immune-related genes such as those encoding triggering receptor expressed on myeloid cells 2 and membrane spanning 4-domains A6A and milder changes in insulin/IGF1 signalling. The precuneus in AD was also characterised by changes in vesicle secretion and downregulation of the interneuronal subtype marker, somatostatin. The ‘early’ AD transcriptome is characterised by perturbations in synaptic vesicle secretion on a background of neuroimmune dysfunction. In particular, the synaptic deficits that characterise AD may begin with the somatostatin division of inhibitory neurotransmission.

## Introduction

Alzheimer’s disease (AD) is the most common neurodegenerative disorder and is predicted to become the most impactful health issue for Western countries by 2050 due to an ageing population and the lack of disease-modifying treatments^1^. AD commonly manifests clinically in the eighth decade of life but imaging studies suggest that the disease develops at least 20 years before symptoms are detected^2^. The neuropathology of AD is characterised by widescale atrophy, regional neuronal loss, gliosis and two pathognomonic entities; extracellular plaques, made up primarily of peptides called beta-amyloid (Aβ) and neurofibrillary tangles (NFT) made up chiefly of filaments of hyperphosphorylated microtubule-associated protein tau (tau). A pathological diagnosis is based on a qualitative assessment of NFTs, diffuse and neuritic plaques that prioritises the spread of these entities rather than the degree of pathology in any particular region. As both tau and Aβ pathology commonly occur in aged non-demented individuals^3^ a diagnosis of AD is probabilistic with no clear threshold of pathology that, above which, dementia is conferred.

Given that neuronal loss is synonymous with symptoms in AD and irreversible, any disease-modifying drug needs to be implemented before such loss occurs. The popular amyloid (cascade) hypothesis (ACH) states that the pathogenesis of common forms of AD is precipitated by accumulation of neurotoxic and soluble Aβ oligomers in the brain parenchyma^4^. It further proposes a linear process where Aβ oligomers cause neuronal dysfunction that ultimately results in NFT formation, neuron dysfunction and death. ACH is based on the phenotypic similarities between rare familial and sporadic forms of AD and supported by positive emission tomography (PET) studies that show Aβ load in presymptomatic individuals^5^. In particular, PET imaging of asymptomatic mutation carriers suggests that the Aβ accumulation in the precuneus region is a key early event in disease progression^6^. It is unclear how amyloid and tau are in this cascade but one hypothesis is that Aβ attenuates central insulin signalling and AD has been called ‘Type 3 Diabetes’^7,8^. The subsequent downregulation of the associated PI3K/AKT pathway is proposed to lead to excessive activity by major tau kinases, primarily glycogen synthase Kinase 3β (GSK3β) that is normally inhibited by insulin to enable glycogen synthase activity in cells^9^.

The findings from pathological studies in AD do not entirely support the linear process proposed by the ACH. Aβ pathology begins widely in the neocortex, particularly the ventral neocortex, before spreading to subcortical regions, the brainstem and cerebellum^10^. Aβ pathology shows more variation between cases than tau pathology and it plateaus in many regions early in the disease^11^. Staging studies suggest that tau pathology has a more predictable pattern, beginning in the transentorhinal cortex before spreading through the limbic system and into the neocortex with relative sparing of the primary cortices such as the primary visual cortex^12^. There are various forms of tau or neurofibrillary pathology such as dystrophic neurites^13^, neuritic threads and astroglial pathology^14^ but staging schema concentrate on neurofibrillary tangles (NFTs). AD symptoms are correlated with irreversible neuronal loss and this in turn is correlated to the spread of NFTs rather than Aβ pathology^12,15^.

There are also a number of challenges in understanding the course of events within the ACH. Firstly, the long prodrome and disease duration would be consistent with a subtle pathogenic mechanism^16^. Second, mechanistic studies are best performed in animal models, but the human brain is more complex than any other organ relative to that found in lower mammals. In particular, the human brain transcriptome correlates closely with advanced human cognition^17^. As expected, there is little concordance between brain transcriptomic data from human AD brain and AD animal models^18,19^.

Post-mortem tissue, the only consistently available source of human brain tissue, appears unsuitable for mechanistic studies. In neurodegenerative diseases, the most severely affected regions of the brain display considerable neuronal loss^20^, making it challenging to interpret how case–control differences in gene expression from these areas relate to the disease process. A possible compromise is to utilise regions that are affected by the disease, with plaques and, or NFTs, but yet to experience neuronal loss at post-mortem. A putative scenario would see ‘sick’ neurons contributing maximally to the bulk tissue AD transcriptome. In reality, this model is likely to focus on tau-related pathomechanisms given its more predictable spread across the neocortex. By comparing areas mildly and moderately affected by tau pathology within the same AD brains, as well as traditional case–control comparisons, the natural history of the disease might be modelled^21^.

The development of new pre-symptomatic diagnostics and therapeutics in AD is hindered by our lack of understanding of the events promoting and connecting the various stages of the ACH^22^. We hypothesize that early pathogenic changes can be determined from observing gene expression profiles in less severely affected cortical regions of the AD brain at post-mortem. Here we report the pathomechanisms related to early tau deposition from comparing the moderately affected precuneus (PREC) to the mildly affected primary visual cortex (VIC).

## Methods

### Tissue

This study was carried out following ethical approval from The University of Sydney’s Human Research Ethics Committee (HREC#2012/161) with all experiments with human tissue performed in accordance with the Declaration of Helsinki. De-identified tissue was supplied by New South Wales Brain Banks (NSWBB) including the Sydney Brain Bank at Neuroscience Research Australia and the New South Wales Brain Tissue Resource Centre (NSWBTRC) at The University of Sydney following approval from their Scientific Advisory Committee. Informed consent was previously obtained from the participants (or next-of-kin at time of death) by NSWBB, who also provided information on age, gender, dementia status (yes/no) clinical dementia rating (CDR; non-demented = 0: MCI = 0.5; demented > 0.5), AD pathological diagnosis according to latest ‘ABC’ criteria^3^, cause of death, *post-mortem* interval (PMI), and brain pH (Supplementary Table 1 for details with RNA sequencing samples in bold type). 10 μm formalin-fixed paraffin-embedded (FFPE) sections and 100 mg frozen tissue from the contralateral hemisphere of the VIC and PREC were obtained from 26 cases and 22 age-, gender- and APOE ε4-matched neurologically normal controls.

### RNA-Seq

Our overall aim was to explore the plausibility of using differentially affected areas of the *post-mortem* AD brain to model disease progression. As a ‘proof of concept’ study five cases and five controls were selected for RNA sequencing (RNA-Seq) following matching for age, gender, APOE ε4 genotype and RNA quality (RNA integrity number; RIN). RINs had been derived from the superior temporal gyrus (STG) of all donors for a previous study^23^. All cases had been designated with high likelihood of AD (‘ABC’ score) according to the latest pathological diagnostic criteria and all were Braak stage VI^3^ (Supplementary Table 1 for full clinical and pathological data). RNA was extracted from 30 mg frozen tissue from the PREC and VIC using TRIzol (Life Technologies, Thermo Fisher Scientific, Carlsbad, CA 92008, USA) including an on-column DNase I treatment. 500 ng of RNase H ribodepleted total RNA was used for library preparation with the TruSeq Stranded Total RNA Sample Prep Kit (Illumina, USA). Mixes of External RNA Controls Consortium (ERCC) Spike-Ins (Thermo Fisher Scientific) with different molar ratios were added to control (Mix 1) and AD samples (Mix 2). The libraries were sequenced using the Illumina HiSeq2500 at the Kinghorn Centre for Clinical Genomics, Garvan Institute of Medical Research, Sydney, Australia.

### RNA-Seq data analysis

All data analyses were carried out within the R project environment as previously described^24^. Reads were mapped to GRCh38.p5 reference genome using STAR (version 2.5.1a)^25^. Known GENCODE genes (version 24) were quantified by RSEM (version 1.3.0)^26^. The complete code and supplementary quality metrics can be found at: https://github.com/gboris/AD_RNA-Seq. Trimmed mean of M values (TMM) normalization and filtering according to abundance of reads in the either case or control, were carried out in edgeR^27^. An exploratory ANOVA of all RNA-Seq data (both regions), based on all filtered normalised gene counts, was used to identify potential covariates for inclusion in the subsequent differential gene expression analysis for each region (Additional Fig. 1). Status, region, age, gender, post-mortem interval (PMI), brain pH (occipital lobe), and STG-RIN^23^ were included as covariates in the model to derive differentially expressed genes (DEG) with ‘edgeR’^28^. Significance was set at a false discovery rate (FDR)-adjusted *p* value ≤ 0.05. Gene enrichment analysis was performed using various databases including: Gene Ontology (GO) sets of molecular functions (MF), biological processes (BP), and cellular components (CC). General concordance between gene associations in the two regions was explored by correlation analysis fold-changes.

### Quantitative neuropathology

The VIC and PREC of all 26 cases and 22 controls were quantified for tau (AT8-tau) -immunopositive neurons [AT8-tau monoclonal antibody (1:500, #MN1020, Thermo Fisher Scientific, Rockford, IL 61105, USA); Phospho-Tau (Ser202, Thr205)], the areal fraction of Aβ immunopositivity [Beta Amyloid, 1–16 (6E10) monoclonal antibody (1:500, #SIG-39320-200, BioLegend, San Diego, CA 92121, USA)] and the total number of cortical neurons. Briefly, immunopositive neurons or all cortical neurons were counted manually in three cortical strips (cortical surface to grey matter/white matter boundary) from a 10 μm section of either PREC or VIC with an eyepiece graticule. AT8-tau immunopositive (+ ve) and cortical neuron counts were converted into cells per mm^2^ value by dividing total neurons by the number of graticules across the three strips, while Aβ immunopositivity was calculated as an areal fraction. The mean density/areal fraction for each individual region was calculated and normalised for potential atrophy by multiplying by a correction factor (mean cortical width/mean cortical width of gender-matched controls) (Supplementary Table 1 for full quantitative neuropathology data).

### RNA-Seq validation—droplet digital PCR

RNA was further isolated from 30 mg human brain tissue of all 26 cases and 22 controls including additional tissue from those individuals previously sampled for RNA-Seq. Here RNA was extracted using TRIzol reagent in combination with the PureLink RNA Isolation Kit (Life Technologies, Thermo Fisher Scientific) including an on-column DNase I treatment. RNA was eluted with RNase free water and stored at − 80 °C until use. cDNA was synthesised using the sensiFAST cDNA kit (Bioline, Sydney, NSW 2015, Australia) and diluted to 100 ng prior to amplification by droplet digital polymerase chain reaction (ddPCR). Transcript abundance was measured using the Bio-Rad QX200 Droplet Digital PCR System (Bio-Rad Life Science, Hercules, California 94547, USA) at the Molecular Biology Facility, Bosch Institute, The University of Sydney. Primers for genes of interest (GOI) were designed using PrimerBlast (https://www.ncbi.nlm.nih.gov/tools/primer-blast/) and manufactured by Integrated DNA Technologies Australia Pty Ltd (Table 1 for sequence information). A preliminary experiment in the five RNA-Seq controls only was carried out with *SST* and four potential housekeeping genes: glyceraldehyde-3-phosphate dehydrogenase (*GAPDH*), hydroxymethylbilane synthase (*HMBS*), hypoxanthine phosphoribosyltransferase 1 (*HPRT1*) and glial fibrillary acidic protein (*GFAP*) as previously described^23^. All assays were optimised such that there was a clear differentiation of positive and negative droplets as per manufacturer’s instructions. *GAPDH* was selected as the reference genes based on a similar positive correlation with RIN in the controls to *SST*, to minimise potential premortem effects on disease-specific differential expression as previously described^23^. ddPCR was then extended to the full cohort with cDNA at 100 ng except for GAPDH (12.5 ng) with data normalised as GOI/*GAPDH.* An ANOVA was used to determine group differences for both regions. As GOIs, other than *SST,* did not necessary correlate with RIN in the controls similarly to *GAPDH*, F ratios were also adjusted for age-, gender- and region-specific RIN by logistic regression. Correlations between normalised GOI expression and neuropathology (AT8-tau positive neurons, areal Aβ fraction and residual neurons) were also explored in the cases only and adjusted as above. All statistical analyses were carried out in JMP10.0.0 (SAS Institute Inc., Cary, North Carolina, USA).

**Table 1:** Droplet digital PCR primer details.

Gene name	Primer sequences	Primer concentration (nM)
Somatostatin	Forward 5′-ACCCCAGACTCCGTAGTTT-3′ Reverse 5′-AGTACTTGGCCAGTTCCTGCT-3′	125
Insulin receptor	Forward 5′-GGACCAGGCATCCTGTGAAA-3′ Reverse 5′-GGGCCTCTTTGTAGAACAGCA-3′	125
Insulin-like growth factor 1 receptor	Forward 5′-AGGAATGAAGTCTGGCTCCG-3′ Reverse 5′-CCGCAGATTTCTCCACTCGT-3′	125
Glyceraldehyde 3-phosphate dehydrogenase	Forward 5′-AAATCAAGTGGGGCGATGCT-3′ Reverse 5′-CAAATGAGCCCCAGCCTTCT-3′	50
C-X-C motif chemokine receptor 4	Forward 5′-GAGTGCTCCAGTAGCCACC-3′ Reverse 5′-GCCCATTTCCTCGGTGTAGT-3′	50
Membrane spanning 4-domains A6A	Forward 5′-GCTGATTTGCACTCTGCTGG Reverse 5′-GCAGGAAAAGTACACTCCCAGG-3′	50
Triggering receptor expressed on myeloid cells 2	Forward 5′-TGCTGGCAGACCCCCTG-3′ Reverse 5′-GAAGGATGGAAGTGGGTGGG-3′	50
Parvin gamma	Forward 5′-AAATGCTGCACAACGTCACC-3′ Reverse 5′-AGGCAGTGAGGGTCAATTCG-3′	50
Solute carrier family 7 member 2	Forward 5′-CCATTTTCCCAATGCCTCGTG-3′ Reverse 5′-GAAAGGCCATCAAAGCTGCC-3′	50

### RNA-Seq validation—ROSMAP study data

Permission was obtained to access the Religious Orders Study and Memory and Aging Project (ROSMAP) study data from the Rush Alzheimer’s Disease Center and hosted by Sage Bionetworks—Synapse system (https://www.synapse.org/#!Synapse:syn3219045). RNA-Seq data was from the dorsal lateral prefrontal cortex of participants who had undergone comprehensive neuropsychological assessment to assign cognitive status as previously described^29^. ROSMAP participants were classified as cognitively normal, having mild cognitive impairment or demented. The ROSMAP study is unique in that quantitative neuropathological examination has been performed across multiple brain regions^30^ in combination with the semi-quantitative and probabilistic standard AD diagnostic criteria which classifies individuals with low, intermediate or high likelihood that their dementia is primarily due to AD^31^. A whole genome co-expression network analysis of the RNA-seq data on 478 individuals has been previously reported^32^ but was re-analysed here by comparing the most disparate groups—non-demented controls with no or low AD pathology (n = 117) versus demented individuals with intermediate or high AD pathology (n = 195)—using the same differential expression method described above.

### Comparison with GWAS data

Seyfried and colleagues previously described a strategy for determining if protein products of GWAS targets are enriched in gene expression data^33^. Using data from International Genomics of Alzheimer’s Project^34^ they used the gene set analysis program called MAGMA^35^ to derive 1234 GWAS (AD risk) genes. A Chi-square test was used to determine if the DEGs in the AD-PREC or AD-VIC were significantly over-represented among their AD risk genes.

### Ethics approval and consent to participate

Ethics approval for the use of de-identified post-mortem human tissue has been described in the Methods section, Paragraph 1.

## Results

RNA sequencing (RNA-Seq) was carried out on samples from the PREC and VIC brain regions of five AD cases and five controls matched for age, gender, APOE genotype and RIN and selected from a previously described cohort^23^ (Table 2 and Supplementary Table 1 for full data).

**Table 2:** Demographics and clinicopathological information.

Mean (S.D.)	AD (n = 5)	Control (n = 5)	*p* value
Age (years)	76 (14)	78 (9)	0.8
Post-mortem interval (hours)	11 (12)	18 (18)	0.3
Brain pH (occipital lobe)	6.5 (0.2)	6.6 (0.2)	0.1
RIN (STG for matching)	6.9 (0.3)	6.9 (0.4)	0.9
RIN (PREC)	5.2 (0.3)	5.5 (0.3)	0.5
RIN (VIC)	5.3 (0.2)	5.3 (0.2)	1

All AD cases had been previously classified as Braak stage VI according to current diagnostic criteria^3^ although one case (IS18), a 100 old female, had a relatively low tau load in her PREC (7.1 immunopositive cells/mm^2^) (Supplementary Table 1 for all details). The regional pathology in the AD cases was consistent with the expected pattern of tau pathology across the neocortex with relative sparing of VIC and generalised Aβ deposition across the entire neocortex (including VIC and PREC) early in the disease course^12,15^. Neither region showed any difference in neuronal density between cases and controls, but both were characterised by higher Aβ areal fractions in the cases (Table 3). In contrast, only the PREC of AD cases (AD-PREC) had more AT8-tau + ve neurons compared to controls. Among the cases, Aβ areal fractions were similar in both regions (*p* = 0.5) but AT8-tau + ve neurons trended towards being higher in the PREC (*p* = 0.08) (Supplementary Table 1 for all quantitative neuropathology data; RNA-Seq samples in bold type).

**Table 3:** Quantification of AD neuropathology in the RNA-Seq cohort.

Mean (S.D.)	AD (n = 5)	Control (n = 5)	*p* value
PREC—AT8-tau + ve neuron density (mm^2^)	29 (13)	0	0.001
PREC—Areal Aβ fraction	0.09 (0.05)	0	0.006
PREC—Neuronal density (mm^2^)	303 (31)	269 (64)	0.31
VIC—AT8-tau + ve neuron density	13 (13)	0	0.06
VIC—areal Aβ fraction	0.07 (0.05)	0	0.01
VIC—neuronal density	306 (35)	262 (74)	0.26

	PREC	VIC	*p* value
**Inter-regional comparisons (cases only)**			
Areal Aβ fraction	0.09 (0.05)	0.07 (0.05)	0.5
AT8-tau + ve neuron density	29 (13)	13 (13)	0.08

### RNA sequencing

**Figure 1:**
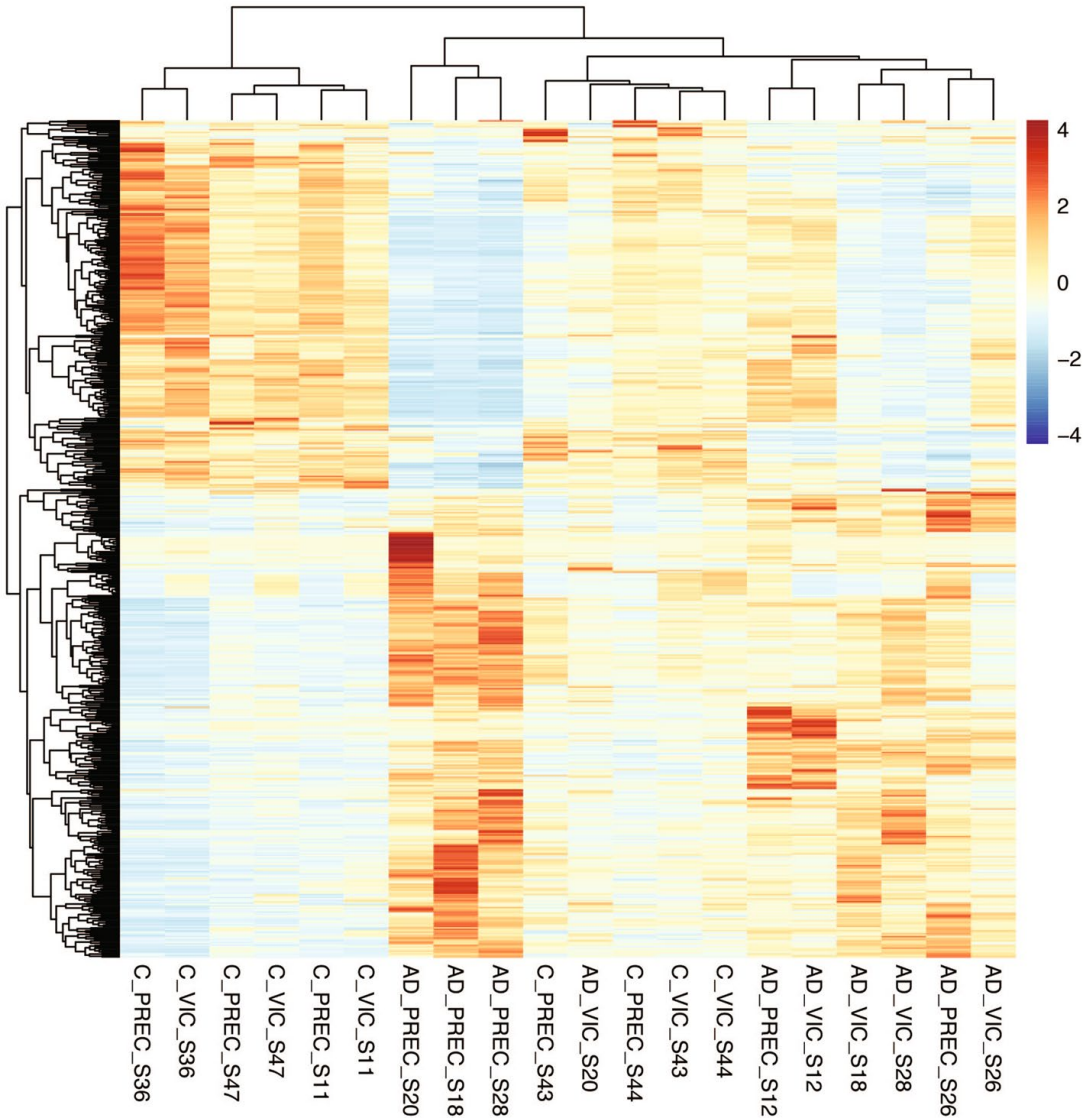
Hierarchical clustering of samples. A heatmap shows the clustering of the 20 Alzheimer’s disease (AD) and neurologically normal control (control) brain tissue samples based on their differential gene (transcript) expression (z-values). Samples are from the primary visual cortex (VIC) and precuneus (PREC); regions that are mildly- and moderately affected by tau pathology respectively in the AD brain at post-mortem. The colour scale shows transcripts that are upregulated (red) or downregulated (blue) relative to mean expression of all samples. Four control samples (PREC and VIC regions from IS43 and IS44) cluster with the cases.

RNA sequencing was carried using 125 bp paired-end approach at an average sequencing depth of 25 million reads per sample. Transcripts that had less than five reads in two or less samples were filtered out prior to differential expression analysis. In order of magnitude data variance was due to: RIN, age, brain region, pH, gender and then disease status (Additional Fig. 1). Excluding region, all these factors were included in linear regression models to generate the DEGs for the VIC and PREC. Hierarchical clustering of the DEGs from both regions demonstrated that control and case samples largely segregated with each other (Fig. 1). Two controls, IS43 and IS44, segregated with the cases and this could not be explained by a common APOE genotype (ε3/ε4 and ε3ε3 respectively) or other available clinical data ( Supplementary Table 1 for full details).

### Precuneus (PREC)

**Figure 2:**
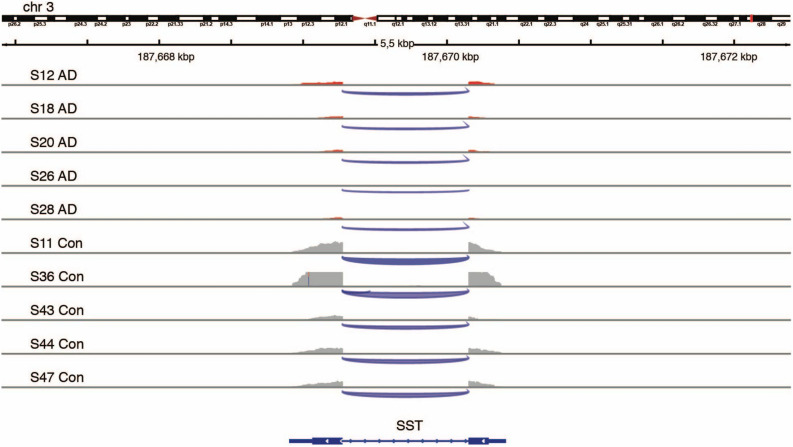
Downregulation of somatostatin gene expression in the precuneus of Alzheimer’s disease brains. An Integrative Genomics Viewer (IGV)-derived image shows the comparative levels of reads from the two-exon somatostatin (SST)-encoding gene within the precuneus region of post-mortem brains from four Alzheimer’s disease (AD) cases (upper tracks) and four controls (Con; lower tracks). (IGV ver. 2.3.91; Broad Institute).

The AD-PREC was characterised by 559 DEGs (462 protein -oding) (FDR ≤ 0.05; Supplementary Table 2). The ERCC subgroup A transcripts that had been spiked-in to the AD samples at a fourfold molar ratio were prominent among the top DEGs and removed from further analysis. The top ten most significant DEGs in the AD-PREC included protein-coding transcripts: immunoglobulin kappa constant (*IGKC*), the cationic amino acid transporter (*SLC7A2*), parvin gamma (*PARVG*) and membrane-spanning 4-domains, subfamily A, member 6A (*MS4A6A*), a probable chemosensor expressed by microglia^36^ (Table 4). *SST*, encoding the interneuron subtype marker somatostatin, was also downregulated in AD-PREC (ranked 14th, log FC = − 2.2, FDR adjusted *p *value = 6.9 × 10^–6^ (Fig. 2) consistent with immunohistochemical deficits in the AD brain^37^.

**Table 4:** Top 10 most differentially expressed genes in the AD-PREC.

Rank	GENCODE #	Gene symbol	Gene name	Log FC	*p* value (FDR adjusted)
1	ENSG00000185710	Non-coding	SMG1P4; SMG1 pseudogene 4	− 6.4	1.29E−19
2	ENSG00000226259	Non-coding	GTF2H2B; General transcription factor IIH subunit 2B	4.6	1.17E−12
3	ENSG00000275954	*TBC1D3F*	TBC1 domain family member 3F	− 10.5	2.13E−09
4	ENSG00000003989	*SLC7A2*	Solute carrier family 7, member 2	1.8	2.86E−09
5	ENSG00000138964	*PARVG*	Parvin gamma	1.4	3.93E−07
6	ENSG00000110077	*MS4A6A*	Membrane-spanning 4-domains, subfamily A, member 6A	1.4	1.27E−06
7	ENSG00000275830	Non-coding	AL355974.2	2.1	1.43E−06
8	ENSG00000211592	*IGKC*	Immunoglobulin kappa constant	3.9	1.43E−06
9	ENSG00000235833	Non-coding	AC017099.1	− 4.1	1.89E−06
10	ENSG00000152049	KCNE4	Potassium voltage-gated channel subfamily E regulatory subunit 4	1.6	2.2443E−06

Up- and down-regulated genes were combined for gene ontology (GO) analyses and this revealed two major themes among the enriched Biological Pathways (GO-BP): exocytosis and immune function (Table 5; Supplementary Table 3 for full list). Genes involved in exocytosis included *STXBP2*, encoding syntaxin binding protein 2 (log FC = 1.0, adj. *p* value = 0.02) and *NSF,* encoding protein N-ethylmaleimide-sensitive factor (log FC = − 1.1, adj. *p* value = 0.006) that are involved in SNARE complex assembly and disassembly respectively.

**Table 5:** Overrepresented gene ontology biological pathways in the AD-PREC.

GO term	Biological pathway	*p* value
GO:0006887	exocytosis	1.6E−12
GO:0032940	secretion by cell	6.2E−12
GO:0045055	regulated exocytosis	4.3E−11
GO:0046903	secretion	6.4E−11
GO:0042119	neutrophil activation	5.8E−09
GO:0002274	myeloid leukocyte activation	6.2E−09
GO:0036230	granulocyte activation	7.5E−09
GO:0043312	neutrophil degranulation	1.1E−08
GO:0002283	neutrophil activation involved in immune response	1.2E−08
GO:0006810	transport	2.1E−08

### Primary visual cortex (VIC)

**Figure 3:**
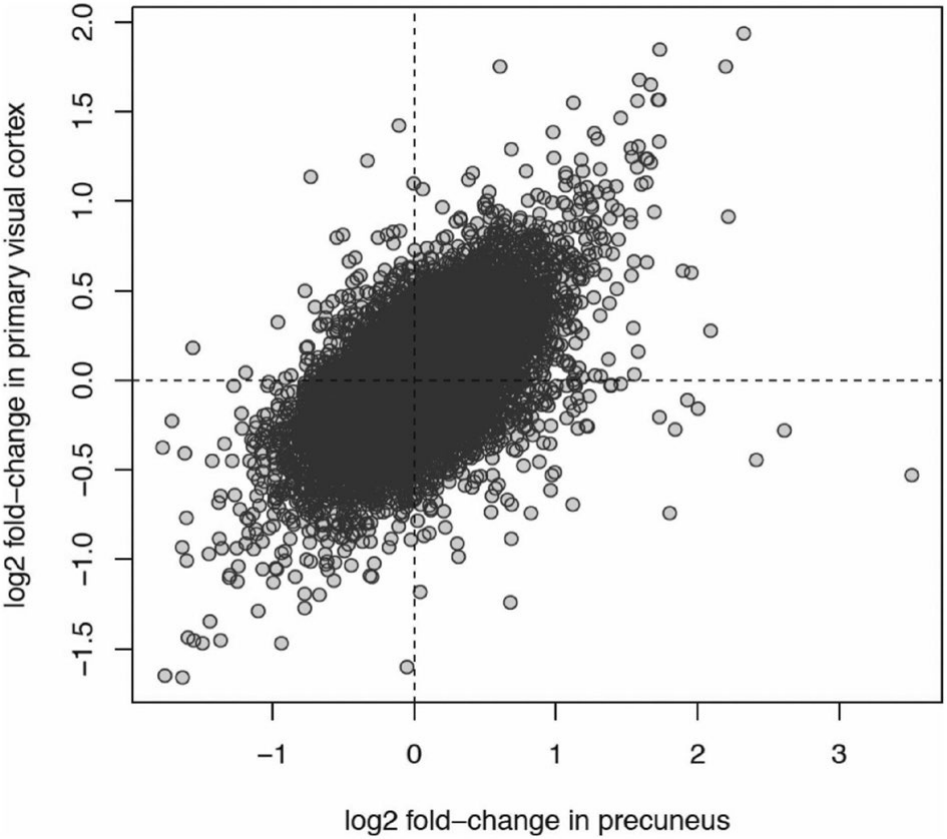
Correlation between Alzheimer’s disease-associated genes in the precuneus versus the primary visual cortex. A scatter plot displays the positive correlation between differential expression of all transcripts in Alzheimer’s disease and control post-mortem brains for the primary visual cortex (y-axis) versus the precuneus (x-axis). Each data point is a gene. The x-axis is the relative log2 fold-change in mean expression between AD and control samples for the precuneus region. The y-axis is the relative log2 fold-change in mean expression between AD and control samples for the primary visual cortex region. A gene with a positive fold-change has higher expression in AD samples. Genes in the upper-right and bottom-left quadrants of the plot have concordant changes in expression between AD and controls. The correlation between the fold-changes in both regions is r^2^ = 0.35.

There were 71 total and 44 protein-coding DEGs in the AD-VIC (adjusted *p* value < 0.05 Table 6; Supplementary Table 4 for full list). 40/71 DEGs in the AD-VIC brain region were differentially expressed in the same direction as in the AD-PREC suggesting similar responses to the presence of AD pathology. Overall, there was a high correlation between the direction of associations of all genes in the AD-PREC versus the AD-VIC (r^2^ = 0.35) (Fig. 3). 27/462 protein coding genes were in common between the two regions including *TBC1D3F*, encoding TBC1 domain family member 3F, *RPS26* and *CD207*. There is little known about the putative oncogene *TBC1D3F,* while *CD207*, a receptor on antigen-presenting Langerhans cells in the skin, has very low expression in the brain. Other DEGs here included the microglial *PARVG* and *MS4A6A* ranked 5th and 6th among the PREC DEGs respectively.

**Table 6:** Top 10 most differentially expressed genes in the AD-VIC.

Rank	GENCODE #	Gene symbol	Gene name	Log FC	*p* value (FDR adjusted)
1	ENSG00000185710	Non-coding	SMG1 pseudogene 4	− 6.3	4.3E−18*
2	ENSG00000275954	*TBC1D3F*	TBC1 domain family member 3F	− 13.3	3.1E−15*
3	ENSG00000205632	Non-coding	LINC01310	− 2.0	2.6E−07
4	ENSG00000197728	*RPS26*	ribosomal protein S26	− 1.2	3.5E−07
5	ENSG00000226259	Non-coding	General transcription factor IIH subunit 2B (pseudogene)	3.5	3.5E−07^a^
6	ENSG00000275830	Non-coding	AL355974.2	2.1	7.8E−06
7	ENSG00000116031	*CD207*	CD207 molecule, langerin	− 5.8	1.4E−04
8	ENSG00000250770	Non-coding	AC005865.2	− 1.5	1.5E−04
9	ENSG00000254269	Non-coding	CTD-2281E23.2	− 1.9	1.5E−04
10	ENSG00000134201	*GSTM5*	Glutathione S-transferase mu 5	− 1.4	1.6 E−04

^a^DEG in AD-PREC.

Four immune-related GO-BPs in the AD-PREC were similarly among the top ten overrepresented pathways seen in the AD-VIC (Table 7; Supplementary Table 5 for full list).

**Table 7:** Top 10 overrepresented gene ontology biological pathways in AD_VIC.

GO term	Biological Pathway	*p* value
GO:0002252	Immune effector process	1E−06
GO:0006952	Defense response	4E−06
GO:0050727	Regulation of inflammatory response	2E−05
GO:0006955	Immune response	6E−05
GO:0043312	Neutrophil degranulation	1E−04
GO:0002283	Neutrophil activation involved in immune response	1E−04
GO:0070613	Regulation of protein processing	1E−04
GO:0042119	Neutrophil activation	1E−04
GO:0002446	Neutrophil mediated immunity	1E−04
GO:1903317	Regulation of protein maturation	1E−04

With a 2.5-fold greater tau load compared to the AD-VIC, it was hypothesized that AD-PREC DEGs would include upregulated tau kinases and downregulated tau phosphatases^38^. In particular, AD as a putative form of ‘Type 3 diabetes’^7^ would be consistent with impaired central insulin (PIK3-AKT) signalling and increased glycogen synthase kinase 3beta (GSK3β) levels^39^. Contrary to these hypotheses, *CDK5R2*, encoding a neuron-specific activator of the tau kinase cyclin dependent kinase 5, was downregulated, while the genes, phosphoinositide-3-kinase adaptor protein 1 (*PIK3AP1*) and phosphoinositide-3-kinase regulatory subunit 5 (*PIK3R5*)*,* whose products promote PI3K activity, were both upregulated. Similarly, *INSR*, encoding the insulin receptor, and *IGF1R*, encoding the major IGF1 receptor, approached higher expression in the AD-PREC (unadjusted *p* values = 0.06 and 0.01 respectively).

### Non-coding RNAs

97/559 DEGs in the AD-PREC and 27/71 in AD-VIC were long non-coding transcripts included two of most significant DEGs in both regions: SMG1 pseudogene 4 (*SMG1P4*; ENSG00000185710; logFC = − 6.4; FDR = 1.3 × 10^–19^) and general transcription factor IIH subunit 2B (GTF2H2B; pseudogene; ENSG00000226259; logFC = 6.4; FDR = 1.2 × 10^–12^).

### ddPCR replication in a larger cohort

We selected eight protein-coding DEGs from the RNA-Seq study for validation in the full cohort of 26 AD cases and 22 controls: *SST*, *SLC7A2*, *TREM2*, *CXCR4* and *MS4A6A* and *PARVG, INSR and IGF1R.* As for the RNA-Seq cohort there was no difference in neuronal density between cases and controls for either region. However, both regions were characterised by higher Aβ areal fractions and more AT8-tau + ve neurons in the cases (Table 8). Among the cases, Aβ areal fractions were similar for both regions (*p* = 0.5) but AT8-tau + ve neurons were higher in the PREC (*p* = 0.0009) (Supplementary Table 1 for all quantitative neuropathology data; RNA-Seq samples in bold type).

**Table 8:** Quantification of AD neuropathology in the RNA-Seq cohort.

Mean (S.D.)	AD (n = 26)	Control (n = 22)	*p* value
PREC—AT8-tau + ve neuron density (mm^2^)	20 (14)	0	< 0.0001
PREC—areal Aβ fraction	0.07 (0.05)	0.008 (0.008)	< 0.0001
PREC—neuronal density (mm^2^)	276 (56)	283 (63)	0.7
VIC—AT8-tau + ve neuron density (mm^2^)	8 (9)	0	0.0003
VIC—areal Aβ fraction	0.07 (0.05)	0.002 (0.007)	< 0.0001
VIC—neuronal density (mm^2^)	309 (41)	278 (62)	0.06
PREC—RIN	4.1 (0.9)	5.2 (1.0)	0.0001
VIC—RIN	4.2 (0.9)	5.1 (1.2)	0.003

	PREC	VIC	*p* value
**Inter-regional comparisons (cases only)**			
Areal Aβ fraction	0.07 (0.05)	0.07 (0.05)	0.8
AT8-tau + ve neuron density	20 (14)	8 (9)	0.0009

**Figure 4:**
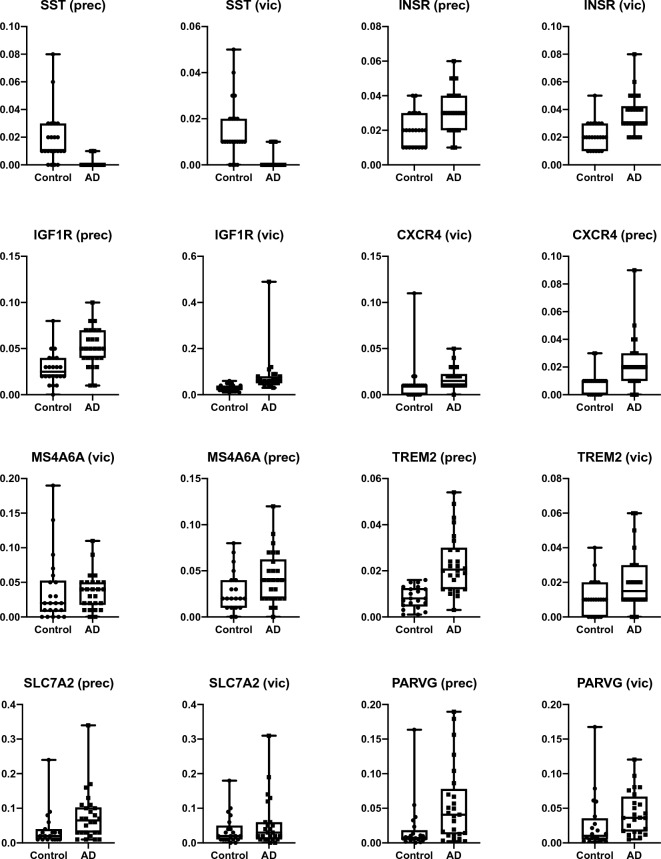
Validation of somatostatin and insulin/IGF1 signalling genes. Histograms show the differential expression, quantified by droplet digital (dd) PCR, of protein encoding genes that were differentially expressed in RNA-Seq: somatostatin (*SST*), insulin receptor (*INSR*), insulin-like growth factor 1 receptor (*IGF1R*), C-X-C motif chemokine receptor 4 (*CXCR4*), membrane spanning 4-domains A6A (*M4A6A*), triggering receptor expressed on myeloid cells 2 (*TREM2*), solute carrier family 7 member 2 (*SLC7A2*), parvin gamma (*PARVG*), in the precuneus (PREC) and primary visual cortex (VIC) of post-mortem brains from 26 Alzheimer’s disease (AD) cases and 22 neurologically normal controls. This includes resampling of all individuals (n = 10) assayed by RNA-Seq.

Unlike the RNA sequencing samples, the mean RIN was significantly lower in the cases in both the PREC (mean RIN 4.1 ± 0.9 v. 5.2 ± 1.0, *p* = 0.0001) and the VIC (AD mean RIN 4.2 ± 0.9 v controls mean RIN = 5.1 ± 1.21; *p* = 0.003) and required normalisation using the reference gene *GAPDH* and further adjustment for regional RIN using logistic regression. *SST* was significantly lower in the AD-PREC (*p* < 0.0001) while *TREM2* (*p* < 0.0001), *MS4A6A* (*p* = 0.0004) and *CXCR4* (*p* = 0.003) were higher in AD cases consistent with the RNA-Seq results (Fig. 4). Similar to RNA-Seq data, *SST* was lower in the AD-VIC (*p* = 0.0001), but neither *MS4A6A* nor *PARVG* were upregulated in the full cohort although *MS4A6A* was highly correlated with age (*p* = 0.001). In contrast to RNA-Seq results, both *INSR* (*p* = 0.001 and *p* = 0.0001) and *IGF1R* (*p* = 0.0002 and *p* = 0.01) were higher in the AD-PREC and AD-VIC respectively (Fig. 4) (Fig. 4). In the AD-VIC, *IGF1R* (r^2^ = 0.35, *p* = 0.002), SST (r^2^ = 0.21, *p* = 0.02) expression was correlated with tau-positive neurons; *PARVG* (r^2^ = 0.25, *p* = 0.02) were correlated with residual neurons and *CXCR4* (r^2^ = 0.28, *p* = 0.007) was correlated with Aβ areal fraction. There were no correlations between AD pathology and GOIs in the AD-PREC.

### RNA-Seq validation—ROSMAP study data

The DEGs from the AD-PREC were compared with a recently published dataset from the Religious Orders Study and Memory and Aging Project (ROSMAP) study. The ROSMAP RNA-Seq data was from the dorsolateral prefrontal cortex from 478 individuals of varying cognitive function and pathological diagnoses including probable AD were originally analysed by a co-expression network technique^32^. Here we restricted the re-analysis to their two most disparate groups: Non-demented controls with no or low AD-type pathology (n = 117) versus demented individuals with intermediate or high AD pathology (n = 195). This revealed 3904 DEGs (3132 protein-coding) of which 203 were among the 462 protein-coding DEGs seen in the AD-PREC here. *SST* was downregulated and *INSR*, *CXCR4*, *SLC7A2* and *NFKBIA* were upregulated, respectively but *IGF1R, TREM2* and *MS4A6A* was not significantly different. The most prominent GO-BPs included ‘positive regulation of nucleic acid-templated transcription’ and ‘neurogenesis’ but there were no immune-related pathways affected.

### Overlap with AD risk genes

Seyfried and colleagues previously described a strategy for determining if protein products of GWAS targets are enriched in gene expression data using the gene set analysis program called MAGMA^33^. They reported1234 that reached MAGMA GWAS significance using International Genomics of Alzheimer’s Project data^34^. A chi-square analysis showed that AD-PREC DEGs were over-represented among their AD risk genes (overlap = 32: *p* = 0.02) but not in the AD-VIC (overlap = 3). Two GWAS targets, *MS4A6A* and *HLA-DRB5*, were in common to both regions (Supplementary Table 6).

## Discussion

Pathological studies suggest that Aβ pathology develops concurrently across most cortical areas and plateaus early in the disease course^10^. In contrast, the severity of tau pathology is known to differ widely across the cortex, sparing the primary cortices^12^, as demonstrated here by mildly-affected VIC versus the moderately-affected PREC. A comparison between such regions both within cases and between cases and controls may reveal the specific expression patterns associated with tau deposition.

### Immune pathways strongly associate with mild AD

The upregulation of immune-related pathways and microglia-expressed genes such as *TREM2* and *MS4A6A,* along with *NFKBIA* (encoding NFKB inhibitor alpha) and *CXCR4* (encoding C-X-C motif chemokine receptor 4) here are consistent with both GWAS and transcriptomic studies^40,41^. Specific gene changes were replicated by ddPCR in the AD-PREC but not the AD-VIC, although *MS4A6A* was correlated with age in the latter.

Similarly AD-PREC DEGs were significantly over-represented among known AD risk genes from GWAS^33^ with immune-related *HLA-DRB5* and *MSA4A6A* also among were among the 45 protein-coding DEGs in the AD-VIC. This overlaps suggests that both *HLA-DRB5* and *MSA4A6A* influence AD pathogenesis through fine tuning of their gene expression levels. The ROSMAP study RNA-Seq data from the prefrontal cortex was mostly confirmatory with RNA-Seq results from the PREC here although *MS4A6A* or *TREM2* were not differentially expressed. In the original gene co-expression analysis of the ROSMAP RNA-Seq the reported ‘microglial module’, that did include *TREM2* and *MS4A6A,* was associated with age but not AD pathology or dementia status^32^. The module most associated with cognitive decline was enriched for genes involved in the “positive regulation of RNA metabolic process”. A similar pathway, ‘positive regulation of nucleic acid-templated transcription’ was the most enriched among the DEGs from our re-analysis of the ROSMAP data here.

This association between immune dysfunction and ageing, rather than AD status is consistent with our previous immunohistochemical studies of microglia morphology in the VIC, the superior frontal gyrus and the inferior temporal gyrus (ITG)^42^. This study, that also included one case here (IS38; Supplementary Table 1 for details) showed that activated microglia in the VIC and SFG, were associated with age but not disease status while the severely affected ITG in AD was characterised by a loss of normal (ramified) microglia. Although not reported, the PREC was also examined and showed the same associations between activated morphologies and age^42^. In contrast, controls with sufficient AD-type pathology to satisfy an intermediate likelihood of disease (according to current diagnostic criteria^3^) did have significantly more activated microglia than controls. Given the similarities in Aβ accumulation between the two regions it seems that disease-specific immune dysfunction may be more related to tau pathology as has been reported elsewhere for *TREM2* and *MS4A6A*^43^. In contrast, the down regulation of *SST*, and upregulation of the insulin/IGF1 signalling pathway appear to be some of the earliest changes in the AD brain, or at least changes that prevent or limit tau pathology.

One of our major interests is to match the molecular findings from frozen post-mortem brain tissue to the immunohistochemical findings gathered from the mirror region in the contralateral hemisphere. This allows disease-specific changes to be further nuanced as either Aβ- or tau-related or related to residual neurons. Here correlations with pathology were only seen in the AD-VIC where the microglial- expressed *CXCR4* was related to Aβ load. *TREM2* and *IGFR1* were associated with residual neurons and with tau pathology respectively but given that most neurons remain in the AD-VIC and tau-positive neurons are rare these correlations may prove to be spurious findings.

### Transcriptomic support for dysfunctional exocytosis

Synaptic dynamics, another major theme from GWAS of AD, was also prominent in the AD-PREC but not AD-VIC^44^. DEGs included *MEF2C* (encoding myocyte enhancer factor 2C)^34^, which normally limits excessive synapse formation, and it was downregulated here. Similarly, DEGs included regulatory components of the SNARE (soluble NSF attachment protein receptor) protein complex, that facilitates membrane fusion of vesicles^45^. *STXBP2,* whose encoded product, syntaxin binding protein 2, docks vesicles to membranes^46^ was increased in AD-PREC, while N-ethylmaleimide-sensitive factor (NSF), a neuronally expressed ATP-ase^36^ involved in disassembling docked vesicles^45^ was reduced in AD-PREC. This is consistent with the increase of extracellular vesicles in AD brain^47^ and increased synaptic activity enhancing tau propagation and tau pathology^48^.

### Genes associated with tau deposition

The major hypothesis tested in this study was that the combination of tau and Aβ pathology in the absence of neuronal loss meant that ‘sick’ or dysfunctional neurons would contribute substantially to the disease transcriptomic signature. A second hypothesis was that the greater tau load in the AD-PREC would result in greater changes in gene expression than those observed in the AD-VIC. More specifically, genes involved in tau hyperphosphorylation and NFT formation would be differentially expressed in the AD-PREC only or to a greater magnitude than seen in the AD-VIC. In support of our second hypothesis there were eightfold more DEGs in the AD-PREC, but on a background of similar changes in gene expression as indicated by the strong correlation between genes associated with AD in both areas. The differential expression of subunits of tau kinases CDK5 and PI3K are potentially consistent with the greater pathological tau load in the AD-PREC, although the direction of change for subunits of CDK5 (down) and PI3K (up) were opposite to what would have been predicted to result in the hyperphosphorylation of tau. In particular, the increase in PI3K components is consistent with increased PI3K/AKT signalling pathway and inhibition of another tau kinase, GSK3β. Alternatively, these changes could represent efforts by residual cells to counter tau-related neurodegeneration. Liang et al.^49^ came to this conclusion based on their microarray analysis of non-NFT bearing cortical neurons from the AD-VIC and five other regions of the AD brain. While they did not investigate the PREC, they reported more DEGs in areas with greater tau and Aβ pathology including entorhinal, hippocampal, middle temporal, superior frontal and posterior cingulate cortices. Specifically, they showed that *CDK5* was downregulated in all regions apart from the AD-VIC.

### Insulin/IGF1 signalling upregulated early

It is not clear how diabetes modifies AD risk but it has been proposed that central insulin resistance in the AD brain, coined type 3 diabetes, leads to increased activity of the major tau kinase, GSK3β^7^. Furthermore, a rat study suggests that IGF1 infusion can rescue Aβ-dependent deficits in the hippocampal somatostatinergic system^50^. Although not surviving correction for multiple comparisons in our RNA-Seq data, *INSR* and *IGF1R,* were upregulated in both regions of the AD brain according to ddPCR. *INSR*, but not *IGF1R*, was also differentially expressed in our re-analysis of the ROSMAP dataset. Yet an upregulation of insulin/IGF1 signalling machinery appears contrary to the idea of central insulin resistance or it represents a compensatory mechanism that is bolstering neuroprotection in areas of the AD brain yet to experience neuronal loss. Indeed a recent review of all *post-mortem* tissue studies favours an increase in PI3K/AKT activity which is consistent with a neuroprotective but ultimately inadequate response to the disease process^39^.

### Somatostatin—back to the future

The downregulation of the gene encoding somatostatin (*SST*) was particularly interesting given that early pathological studies have shown that both mRNA and protein levels were low across the AD brain^37,51,52^. Somatostatin levels were also correlated with the reduction in choline acetyltransferase activity in AD and somatostatin-positive neurites have been reported within neuritic plaques^51^. From this work, somatostatin replacement therapy for AD was suggested in 1991^53^, although it would be the augmentation of cholinergic activity through the use of acetylcholine esterase inhibitors that would ultimately reach the clinic^54^. The lower *SST* levels reported here are also consistent with a recent meta-analysis of microarray data^55^. Somatostatin is expressed by 30% of cortical interneurons, one of the three main GABAergic interneuron types in human cerebral cortex, the others expressing either parvalbumin (40%) or the serotonin receptor 5HT3a (30%)^56^. Experiments in *SST* knock-out mice suggest that somatostatin-positive interneurons in the medial entorhinal cortex synapse with the dendrites of grid cells to maintain periodic spatial firing^57^. Somatostatin-positive interneurons modulate excitatory input to principal neurons and are postulated to play a role in experience-dependent activity. Paradoxically, in an amyotrophic lateral sclerosis (ALS) and frontotemporal dementia (FTD) mouse model, hyperactive somatostatin interneurons led to disinhibition of layer 5 pyramidal neurons and excitotoxicity^58^. The mechanism proposed was through the inhibition of nearby parvalbumin-positive interneurons. This disinhibitory circuit has also been shown to operate in layer 4 of the somatosensory cortex, in contrast to the direct inhibitory role of somatostatin interneurons towards layer 2/3 pyramidal cells^59^. Although there are relatively few quantitative studies describing neuronal loss in the AD cortex the earliest neuronal loss probably occurs in layer 2/3^60,61^. The downregulation of the gene encoding complexin 1 *(CPLX1*) and other molecules involved in GABA neurotransmission such as those encoding the glutamic acid decarboxylases, *GAD1* and *GAD2*, seen in the AD-PREC is consistent a loss of inhibitory input^62^.

### Non-coding RNAs

Two of the three most significant DEGs here were the non-coding transcripts *SMG1P4* and GTF2H2B. Although there are no known brain-specific data on these transcripts, SMG1 is also known as nonsense-mediated mRNA decay-associated PI3K-related kinase suggesting that *SMG1P4* might act in a regulatory capacity^63^ similar to, and potentially affecting the same pathway as, the PTEN pseudogene, *PTENP1*^64^. A downregulation of the *SMG1P4* would reduce SMG1 levels and promote PI3K/AKT activity. Alternatively, given the emerging role of splicing defects in AD including in tau, nonsense-mediated decay of mRNA may be a critical quality control mechanism^65,66^*.* As more information is gained on lncRNAs and incorporated into databases the ‘Pathway analysis’ presented here and similar studies may be transformed.

## Conclusion

These results suggest that ‘early’ responses to Aβ accumulation are relatively well tolerated and characterised by aberrant neuroimmune signalling. This same process is seen in the AD-PREC and AD-VIC with 27/44 protein-coding genes in common between the two regions all differentially expressed in the same direction and to the same degree. However, the presence of greater tau pathology in the AD-PREC is synonymous with an upregulation of vesicle exocytosis and we hypothesize that this represents increased excitatory activity as a result of dysfunction and probable loss of somatostatin-positive, inhibitory interneurons. While GABA-ergic function did not feature among these enriched pathways, *SST,* along with glutamate decarboxylases, *GAD1* and *GAD2*, *NSF* and *CPLX1* were all downregulated in the AD-PREC. Alternatively, the downregulation of *SST* in AD may represent the functional loss of an Aβ-interacting molecule that normally reduces the production of neurotoxic Aβ oligomeric species^67,68^.

The hypothesis tested here was that case–control comparisons across differentially-affected areas of the AD brain that have plaques and NFTs but no, or minor neuronal loss, may allow early pathomechanisms to be elucidated from gene expression data. An alternative interpretation is that rather than being differentially affected by AD these two areas are differentially resistance to neuronal loss. The latter view is supported by the presence of supposedly dementia-stage cored plaques, neurtic plaques and NFTs in the ‘spared’ VIC^13^. If this latter interpretation is correct then the upregulation of immune function and downregulation of GABA-ergic, or specifically somatostatinergic transmission, seen here may be neuroprotective. Certainly our recent work showing that activated microglia are most prominent in non-demented high pathology controls is consistent with the latter^42^.

Differentiating between these two scenarios would be improved by a larger cohort and more regions, although it is not entirely clear whether the probable likelihood of one of these two competing scenarios could be determined by further post-mortem tissue studies alone. For example, the detection of broader and more extensive gene expression changes in severely affected regions of the AD brain will be complicated by relative neuronal loss. The inclusion of tissue from persons with MCI and subjective memory complaints from ‘susceptible areas like the entorhinal cortex as recently reported by Patel and colleagues could also be useful particularly if a neuroprotective interpretation is favoured^69^. In their study, the transcriptome of high pathology controls in regions such as the entorhinal cortex was characterised by “overactivation” of the “glutamate-glutamine cycle” and disruption of the innate immune system and brain energy metabolism. Importantly, therapies based on either (promoting) a neuroprotective or (attenuating) a neurotoxic scenario must be linked to early detection, when augmenting or attenuating these processes can prevent the irreversible neuronal loss that characterises the symptomatic stages of AD.

## Supplementary Information

Supplementary Figure.

Supplementary Table 1.

Supplementary Table 2.

Supplementary Table 3.

Supplementary Table 4.

Supplementary Table 5.

Supplementary Table 6.

## Data Availability

All data supporting the results in found within the Manuscript and accompanying Figures or in Supplementary Tables 1–6. All summary data generated in this study are included in this published article (and supplementary information files). The raw data has been submitted to the Sequence Read Archive (NCBI-SRA; https://www.ncbi.nlm.nih.gov/sra/PRJNA720779). The detailed scripts for all analyses are available at: https://github.com/gboris/AD_RNA-Seq.
