# Author Correction: Defining early changes in Alzheimer’s disease from RNA sequencing of brain regions differentially affected by pathology

**DOI:** 10.1038/s41598-021-97076-y

**Published:** 2021-08-26

**Authors:** Boris Guennewig, Julia Lim, Lee Marshall, Andrew N. McCorkindale, Patrick J. Paasila, Ellis Patrick, Jillian J. Kril, Glenda M. Halliday, Antony A. Cooper, Greg T. Sutherland

**Affiliations:** 1 Brain and Mind Centre and School of Medical Sciences, Faculty of Medicine and Health, The University of Sydney, Camperdown, NSW 2006 Australia; 2 Discipline of Pathology and Charles Perkins Centre, School of Medical Sciences, Faculty of Medicine and Health, The University of Sydney, Rm 6211 Level 6W, Sydney, NSW 2006 Australia; 3 St. Vincent’s Clinical School and School of Biotechnology and Biomolecular Sciences, University of New South Wales, Kensington, NSW 2052 Australia; 4 Garvan Institute of Medical Research, Darlinghurst, NSW 2010 Australia; 5 School of Mathematics and Statistics, Faculty of Science, The University of Sydney, Camperdown, NSW 2006 Australia

Correction to: *Scientific Reports* 10.1038/s41598-021-83872-z, published online 01 March 2021

The original version of this Article contained an error in Table 1, where primer sequences in the gene for “Somatostatin” was incorrect. As a result,

“Forward 5′-ACCCCAGACTCCGTAGTTT-3′”.

now reads:

“Forward 5′-ACCCCAGACTCCGTCAGTTT-3′”.

The original Article has been corrected.

