# Author Correction: Defining early changes in Alzheimer’s disease from RNA sequencing of brain regions differentially affected by pathology

**DOI:** 10.1038/s41598-023-36807-9

**Published:** 2023-06-20

**Authors:** Boris Guennewig, Julia Lim, Lee Marshall, Andrew N. McCorkindale, Patrick J. Paasila, Ellis Patrick, Jillian J. Kril, Glenda M. Halliday, Antony A. Cooper, Greg T. Sutherland

**Affiliations:** 1 Brain and Mind Centre and School of Medical Sciences, Faculty of Medicine and Health, The University of Sydney, Camperdown, NSW 2006 Australia; 2 Discipline of Pathology and Charles Perkins Centre, School of Medical Sciences, Faculty of Medicine and Health, Rm 6211 Level 6W, The University of Sydney, Sydney, NSW 2006 Australia; 3 St. Vincent’s Clinical School and School of Biotechnology and Biomolecular Sciences, University of New South Wales, Kensington, NSW 2052 Australia; 4 Garvan Institute of Medical Research, Darlinghurst, NSW 2010 Australia; 5 School of Mathematics and Statistics, Faculty of Science, The University of Sydney, Camperdown, NSW 2006 Australia

Correction to: *Scientific Reports* 10.1038/s41598-021-83872-z, published online 01 March 2021

The original version of this Article contained an error in the Data availability section, where the link to the raw data in the Sequence Read Archive (NCBI-SRA) was incorrect.

All data supporting the results in found within the Manuscript and accompanying Figures or in Supplementary Tables 1–6. All summary data generated in this study are included in this published article (and supplementary information files). The raw data has been submitted to the Sequence Read Archive (NCBI-SRA; https://www.ncbi.nlm.nih.gov/sra). The detailed scripts for all analyses are available at: https://github.com/gboris/AD_RNA-Seq.

now reads:

All data supporting the results in found within the Manuscript and accompanying Figures or in Supplementary Tables 1–6. All summary data generated in this study are included in this published article (and supplementary information files). The raw data has been submitted to the Sequence Read Archive (NCBI-SRA; https://www.ncbi.nlm.nih.gov/sra/PRJNA720779). The detailed scripts for all analyses are available at: https://github.com/gboris/AD_RNA-Seq.

The original Article has been corrected.

