# Supplementary figures and images for: Defining early changes in Alzheimer’s disease from RNA sequencing of brain regions differentially affected by pathology
Source: Sci Rep. 2021 Mar 1;11:4865. doi: 10.1038/s41598-021-83872-z (PMC7921390; doi:10.1038/s41598-021-83872-z)

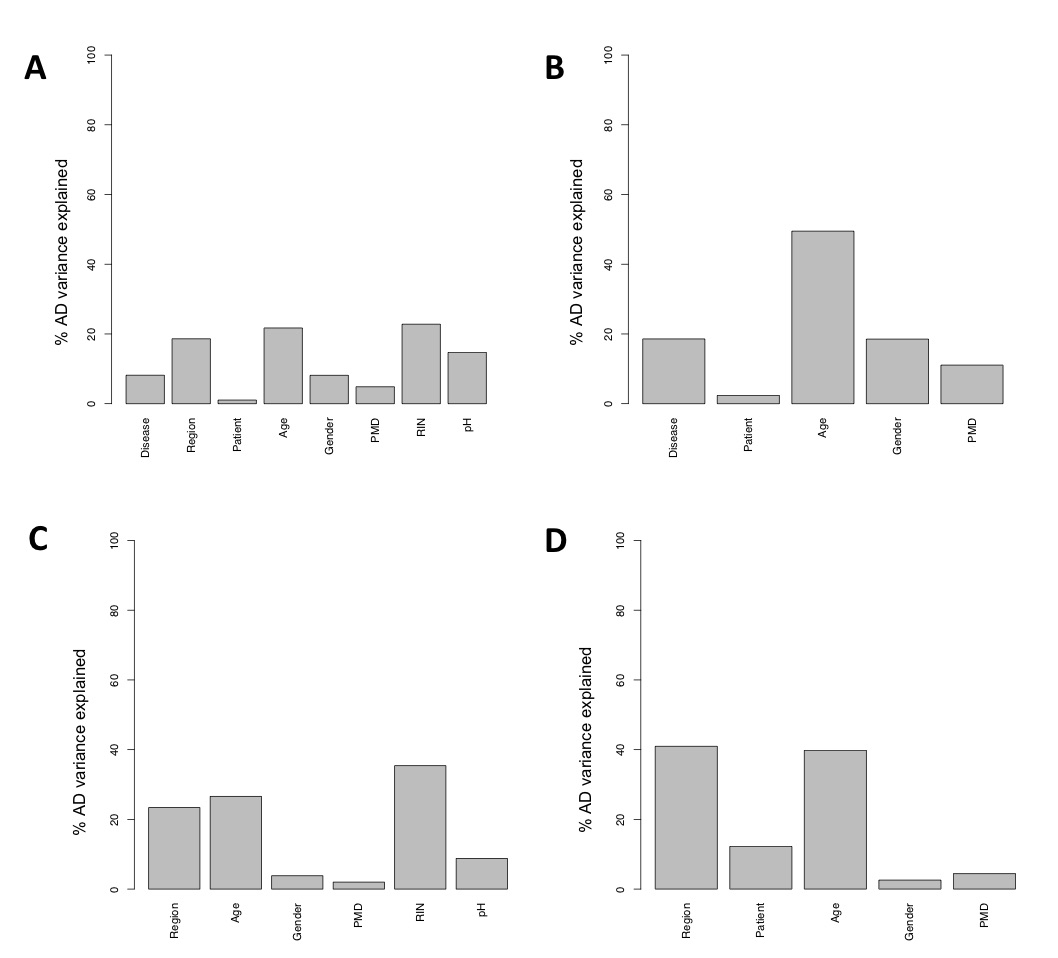

Supplement: Supplementary file 1 — Supplementary Figure. [file 41598_2021_83872_MOESM1_ESM.jpg]
